# 162 A qualitative investigation into causal mechanisms underpinning adherence to physical activity programmes among people with serious mental illness

**DOI:** 10.1093/eurpub/ckae114.037

**Published:** 2024-09-26

**Authors:** Katarzyna Machaczek, Helen Quirk, Hannah Burton

**Affiliations:** Sheffield Hallam University, Sheffield, UK; University Of Sheffield, Sheffield, UK; South West Yorkshire Partnership NHS Foundation Trust, UK

## Abstract

**Purpose:**

Increasing physical activity could reduce the mortality gap experienced by people with serious mental illness (SMI). To be beneficial, physical activity needs to be undertaken regularly over an extended period. This study provides insights into how best to support continued activity for people with SMI by uncovering the underpinning causal mechanisms.

**Methods:**

An international sample of stakeholders (n = 19) were consulted. In-depth, semi-structured interviews were undertaken with i) peer physical activity practitioners (n = 5) who were involved in the design and delivery of physical activity programmes for people with SMI, ii) physical activity leaders (n = 3) and iii) people with SMI who participated in these physical activity programmes (n = 25). Data were analysed using Framework analysis.

**Results:**

Activity that is designed specifically for people with SMI and incorporates structured socialisation opportunities facilitates adherence. Gradual exposure to the group is needed, along with 1-to-1 support incorporating personalised goals. Socialisation opportunities require meticulous planning by the practitioners to carefully curate social interactions that encourage bonding between participants.

Adherence is supported when programmes are scheduled, regular and have no end date. Programmes must address accessibility needs of participants, for example, ensuring that people are supported to get to and from physical activity venues and help them overcome social anxiety. Tailoring support to the fluctuating symptoms of SMI is also imperative.

Qualities of practitioners deemed essential for adherence include soft skills such as the ability to foster warm connections, be accepting and non-judgemental, cultivate mutual respect, not put pressure on participants and encourage autonomous motivation. Mutual compatibility between the participant and practitioner is important and therefore having a choice of practitioners is critical. Adherence is supported when programmes are (co)delivered by practitioners who have lived experience of SMI and who are genuine and vulnerable (they share personal mental health experiences).

**Conclusions:**

A combination of soft skills in practitioners and resource-intensive intervention components determine adherence to physical activity programmes among people with SMI. Ensuring that programmes are adequately planned, supported and resourced with consideration to these mechanisms will be critical for their ability to foster prolonged participation.

**Funding:**

This study was supported by the NIHR Clinical Research Network.

